# Legal vulnerability of orthodontists in professional practice: a cross-sectional study

**DOI:** 10.1590/2177-6709.31.4.e262670.oar

**Published:** 2026-10-12

**Authors:** Isabela Sales PINHEIRO, Cléverson Raymundo Sbarzi GUEDES, Analina Braga APOLINÁRIO, Matheus de Melo PITHON, Isabel Cristina Gonçalves LEITE, Marcio José da Silva CAMPOS, Robert Willer Farinazzo VITRAL

**Affiliations:** 1 Federal University of Juiz de Fora, Dental School, Department of Orthodontics (Juiz de Fora/MG, Brazil).; 2 Federal University of Juiz de Fora, Law School, Department of Substantive Public Law (Juiz de Fora/MG, Brazil).; 3 State University of Southwest Bahia, Dental School, Department of Orthodontics (Jequié/BA, Brazil).; 4 Federal University of Juiz de Fora, Medicine School (Juiz de Fora/MG, Brazil).; 5 Federal University of Juiz de Fora, Dental School (Juiz de Fora/MG, Brazil).

**Keywords:** Orthodontics, Civil liability, Legal vulnerability, Professional practice, Ortodontia, Responsabilidade civil, Vulnerabilidade jurídica, Prática profissional

## Abstract

**Introduction:**

In Brazil, the population’s easy access to the judicial system exposes healthcare professionals, including orthodontists, to the risk of becoming involved in legal disputes for which they are often inadequately prepared.

**Objective:**

In this context, the aim of this study was to assess the legal vulnerability of professionals involved in orthodontic practice and to identify factors associated with professional practice.

**Material and Methods:**

This cross-sectional observational study was conducted using a previously validated questionnaire administered to 247 professionals engaged in orthodontic practice. Participants were classified into low, moderate, and high levels of legal vulnerability. Data were analyzed using descriptive and inferential statistics, including Pearson’s chi-square and Fisher’s exact tests.

**Results:**

The results indicated greater vulnerability among non-specialists, professionals with fewer years since graduation and shorter clinical experience, as well as among those who graduated from private institutions (p < 0.001). In addition, 72.8% of participants believed that patients’ economic status may influence the occurrence of legal claims, and 53.5% reported lacking legal guidance for drafting contracts in their professional practice.

**Conclusion:**

The findings suggest the presence of vulnerabilities related to legal knowledge and professional practices, particularly among less experienced professionals and non-specialists in Orthodontics.

## RESUMO

**Introdução:**

No Brasil, devido a uma facilidade de acesso à justiça pela população, profissionais de saúde como os ortodontistas se veem diante do risco de envolver-se em demandas judiciais para as quais, na maioria das vezes, não se encontram preparados.

**Objetivo:**

O objetivo deste estudo foi avaliar a vulnerabilidade jurídica de profissionais atuantes em Ortodontia e identificar fragilidades relacionadas à prática profissional.

**Material e Métodos:**

Trata-se de um estudo observacional transversal, por meio da aplicação de um questionário previamente validado a 247 profissionais que exercem a atividade de Ortodontia. Os participantes foram classificados em grupos de baixa, moderada e alta vulnerabilidade jurídica. Os dados foram submetidos à análise descritiva e inferencial, utilizando os testes Exato de Fisher e qui-quadrado.

**Resultados:**

A análise dos resultados indicou maior vulnerabilidade entre profissionais sem especialização em Ortodontia, com menor tempo de formação e menor tempo de atuação profissional, bem como entre aqueles profissionais graduados em instituições privadas de ensino (p < 0,001). Além disso, 72,8% dos participantes desse estudo acreditaram que a condição econômica do paciente pode influenciar a ocorrência de demandas judiciais e 53,5% relataram não possuir orientação jurídica para elaboração de contratos durante sua atividade profissional.

**Conclusão:**

Os achados sugerem a existência de vulnerabilidades relacionadas ao conhecimento jurídico e às práticas profissionais, especialmente entre profissionais menos experientes e não especialistas em Ortodontia.

## INTRODUCTION

Concern about the judicialization of issues involving some aspect of the professional activities of liberal practitioners, particularly when linked to claims for compensation arising from consumer dissatisfaction, is not uncommon.^1^ ^,^ ^2^ This is especially relevant in Orthodontics, a highly demanded specialty among patients of different ages, social classes, and, above all, with different treatment goals, whether aesthetic or functional.^3^ ^-^ ^7^ The reasons underlying professional concern about involvement in legal proceedings are numerous, ranging from the orthodontist’s name and social reputation to financial consequences related to indemnification and even the possibility of professional discouragement or reputational harm following a lawsuit.^8^

In Brazil, access to the judicial system is relatively easy for the population, often without the need for legal representation, due to the Federal Constitution of 1988 and, particularly, the strong role played by consumer protection agencies after the enactment of the Consumer Protection Code (CPC), through Law No. 8,078 of September 11, 1990.^9^ ^-^ ^11^ For this reason, orthodontists are exposed to the risk of becoming involved in legal claims for which, in most cases, they are not adequately prepared.^12^ The practice of orthodontics involves extensive debate regarding the legal obligations arising from potential claims for damages, including issues related to objective or subjective liability,^13^ the legal characterization of professional activity as either an obligation of means or an obligation of result,^14^ and the influence of the CPC as a regulatory instrument in the relationship established between patients and healthcare professionals.^15^ ^,^ ^16^

The risks and consequences of legal proceedings for orthodontists are substantial, ranging from exposure to claims to possible financial compensation awards, with potentially multiple repercussions for professional life. For dissatisfied patients, however, the risks are limited, since they are treated as vulnerable parties under the CPC and are often protected by free legal aid.^17^

There is also inappropriate conduct among orthodontists regarding the preparation and storage of clinical records, which may increase professional vulnerability in the event of future legal claims.^12^ ^,^ ^18^

Therefore, the aim of this study was to analyze the application of the questionnaire developed by Guedes et al.,^19^ which assessed the risks and vulnerability of orthodontic professionals to involvement in indemnity-related legal claims, with the purpose of identifying weaknesses in the professional practices of those engaged in Orthodontics.

## METHODS

This cross-sectional observational study was approved by the Research Ethics Committee of the Federal University of Juiz de Fora (CEP/UFJF No. 1,403,552). All participants received information regarding the objectives of the study and provided written informed consent prior to participation. Orthodontic specialists and general dentists engaged in orthodontic practice were considered eligible for inclusion, regardless of sex, age, or years of professional experience. Participants who voluntarily agreed to participate and completed the questionnaire in full were included in the study. Incomplete questionnaires and duplicate responses were excluded from the analysis. The questionnaire developed by Guedes et al.^19^ comprises a total of 53 questions, with five response options arranged according to a Likert scale. It was administered by the researcher, who ensured that participants had understood the instructions and the objectives of the study. For participation in the study, written informed consent forms were collected and thoroughly explained in advance. Participants were recruited both in person and electronically during the data collection period. A total of 247 questionnaire responses were obtained, of which 184 were collected in person and 63 through an online questionnaire administered via the Google Forms platform.

Participants considered eligible for inclusion in the study were orthodontic specialists and general dentists who provided clinical orthodontic care, regardless of sex, age, or years of professional experience. Participants who voluntarily agreed to take part in the study and completed the questionnaire in full were included in the analysis. Incomplete questionnaires and duplicate responses were excluded from the study.

The sample size determination was based on the methodological assumptions adopted in the study conducted by Guedes et al.^19^ for development and validation of the instrument. This approach was grounded in the recommendations proposed by Hair et al.^20^ and Tabachnick and Fidell^21^ for studies involving instrument validation and multivariate analyses, ensuring an adequate sample size for the stability of estimates and appropriate representation of the constructs assessed. Therefore, the sample of the present study was considered adequate for the application of the instrument and for the statistical analyses performed.

The questionnaire included items addressing participants’ sociodemographic and professional characteristics, as well as academic background and knowledge regarding civil liability, documentation practices, contractual relationships, guidance provided to patients, and perception of legal risks inherent to orthodontic practice.

Participants were classified into three groups corresponding to low, moderate, and high legal vulnerability. This classification was performed using cluster analysis, as proposed by Guedes et al.,^19^ based on the response patterns to the qualitative items of the instrument, including undergraduate institution, specialization in Orthodontics, type of institution providing postgraduate training, official registration of the specialty with the professional council, and time since graduation in Dentistry and specialization in Orthodontics. In the instrument used, higher scores corresponded to lower legal vulnerability.

## STATISTICAL ANALYSIS

The data were subjected to descriptive analysis using absolute and relative frequencies. Associations between categorical variables and different levels of legal vulnerability were assessed using Pearson’s chi-square test and Fisher’s exact test, when appropriate. The significance level was set at 5% (p < 0.05). Statistical analyses were performed using SPSS software.

## RESULTS

Table 1 classifies the participants according to their level of vulnerability to legal claims into higher-, moderate-, and lower-risk groups. According to the findings, professionals with more than 7 years of training showed significantly lower vulnerability to risks related to legal actions, corresponding to 4.3% of respondents. In contrast, those with 7 years of training or less exhibited a fivefold higher risk, accounting for 20.7% of respondents (p < 0.001). Time since graduation in Dentistry also influenced professional vulnerability, since participants with 10 years or less since graduation showed higher vulnerability (21.3%), whereas only 2.5% of those with more than 10 years since graduation were classified as higher risk. Regarding specialization, higher vulnerability was observed in 28.7% of non-specialists, compared with only 2.0% of specialists.

**Table 1:** Distribution of participants according to degree of vulnerability to legal claims.

Variables	Degree of vulnerability (clusters)			p-value*
	Low n (%)	Moderate n (%)	High n (%)	
Education				< 0,001
Public	76 (50.0)	69 (45.4)	7 (4.6)	
Private	43 (45.7)	28 (29.8)	23 (24.5)	
Orthodontic specialization				< 0.001
Yes	80 (52.6)	69 (45.4)	3 (2.0)	
No	39 (41.5)	28 (29.8)	27 (28.7)	
Specialization institution				0.070
Public	19 (37.3)	31 (60.8)	1 (2.0)	
Private	52 (59.1)	34 (38.6)	2 (2.3)	
Class entity	10 (71.4)	4 (28.6)	0 (0.0)	
Specialist registration in the council				0.235
Yes	60 (52.6)	53 (46.5)	1 (0.9)	
No	20 (52.6)	16 (42.1)	2 (5.3)	
Care approach method				0.146
In person	92 (50.3)	66 (36.1)	25 (13.7)	
Online	27 (42.9)	31 (49.2)	5 (7.9)	
Time since dental graduation				< 0.001
≤ 10 years	62 (48.8)	38 (29.9)	27 (21.3)	
> 10 years	57 (47.9)	59 (49.6)	3 (2.5)	
Time in orthodontic practice				< 0.001
≤ 7 years	58 (47.9)	38 (31.4)	25 (20.7)	
> 7 years	54 (46.6)	57 (49.1)	5 (4.3)	

*Chi-square test.

Table 2 shows the patient-related factors that participants believed could interfere with the professional-patient relationship and lead to legal claims. Approximately 70% of the participants agreed that the patient’s level of education (76.5%), profession (75.7%), and economic status (72.8%) are factors that can influence the occurrence of a potential lawsuit.

**Table 2:** Dentists’ perception of the influence of patient profile on the professional-patient relationship.

Patient profile	Strongly agree n (%)	Agree n (%)	Neither agree or disagree n (%)	Disagree n (%)	Strongly disagree n (%)
Level of education	91 (36.8)	98 (39.7)	45 (18.2)	11 (4.5)	2 (0.8)
Profession	67 (27.1)	120 (48.6)	43 (17.4)	15 (6.1)	2 (0.8)
Socioeconomic status	70 (28.3)	110 (44.5)	36 (14.6)	22 (8.9)	9 (3.6)

Regarding the respondents’ education and professional training (Table 3), only 13.4% reported having received no guidance on the risks of involvement in legal claims during undergraduate dental education, whereas 6.5% reported receiving no such guidance during postgraduate training. In addition, 80.2% of the participants considered postgraduate training in Orthodontics important both for professional practice and for reducing the likelihood of facing legal claims. Registration of the specialty with professional dental regulatory entities was considered essential by 74.0% of respondents, as shown in Table 1.

**Table 3:** Vulnerability of dentists to lawsuits in orthodontic practice based on legal guidance received during professional training.

Training stage	Always received n (%)	Often received n (%)	Sometimes received n (%)	Rarely received n (%)	Never received n (%)
Guidance during dental school	21 (8.5)	41 (16.6)	84 (34.0)	68 (27.5)	33 (13.4)
Guidance during orthodontic specialization	26 (10.5)	58 (23.6)	108 (43.7)	39 (15.8)	16 (6.5)

With respect to knowledge of the legal implications of professional practice (Table 4), the findings revealed exposure to unnecessary and potentially preventable risks, since a substantial proportion of respondents reported never receiving support from legal professionals, whether for contract drafting (53.4%) or for routine clinical practice (57.5%).

**Table 4:** Dentists’ vulnerability to legal actions in orthodontic practice according to activities performed in professional practice.

Professional activities	Always n (%)	Often n (%)	Sometimes n (%)	Rarely n (%)	Never n (%)
Advertising and disclosure of results	20 (8.1)	27 (10.4)	29 (11.7)	46 (18.6)	125 (50.6)
Evaluation records by another health professional	88 (35.6)	40 (16.2)	52 (21.1)	40 (16.2)	27 (10.9)
Records of joint professional practice	55 (22.3)	26 (10.5)	54 (21.9)	61 (24.7)	51 (20.6)
Legal support for contract preparation	34 (13.8)	13 (5.3)	25 (10.1)	43 (17.4)	132 (53.4)
Legal support as routine professional support	18 (7.3)	8 (3.2)	23 (9.3)	56 (22.7)	142 (57.5)

Table 5 presents the participants’ knowledge regarding legal issues governing professional practice. Only 51.8% of the respondents agreed that orthodontic practice is regulated by the Brazilian CPC, whereas 26.3% expressed no opinion on this matter. Regarding the possibility of criminal or civil liability, 23.9% and 5.2% of respondents, respectively, disagreed that professionals may be held liable in these domains. Furthermore, 57.9% of the participants believed that, in the event of legal proceedings, the burden of proving fault should rest with the dissatisfied patient.

**Table 5:** Participants’ knowledge regarding legal aspects of orthodontic practice.

Legal knowledge	Strongly agree n (%)	Agree n (%)	Neither agree or disagree n (%)	Disagree n (%)	Strongly disagree n (%)
Orthodontics is regulated by the Consumer Defense Code	46 (18.6)	82 (33.2)	65 (26.3)	27 (10.9)	27 (10.9)
Possibility of criminal liability	43 (17.4)	95 (38.5)	50 (20.2)	31 (12.6)	28 (11.3)
Possibility of civil liability	57 (23.1)	124 (50.2)	53 (21.5)	9 (3.6)	4 (1.6)
Burden of proof belongs to the patient	69 (27.9)	74 (30.0)	48 (19.4)	37 (15.0)	19 (7.7)

Table 6 summarizes participants’ professional practices related to legal risk management. Only 15.3% of the respondents reported always or frequently being aware that orthodontic practice may involve an obligation of result, whereas 42.5% stated that they never considered this aspect. Similarly, only 6.5% reported always or frequently maintaining documentary evidence that could support them in the event of legal action, while 75.8% reported rarely or never keeping such records. These findings suggest important gaps in both the understanding of legal principles and the adoption of professional practices aimed at reducing legal vulnerability.

**Table 6:** Professional conduct related to legal risk management in orthodontic practice.

Professional conduct	Always	Often	Sometimes	Rarely	Never
	n (%)	n (%)	n (%)	n (%)	n (%)
Awareness of obligation of result	28 (11.3)	10 (4.0)	52 (21.1)	52 (21.1)	105 (42.5)
Possession of documentary evidence	2 (0.8)	14 (5.7)	44 (17.8)	95 (38.1)	93 (37.7)

With regard to contractual relationships (Table 7), the need for written and individualized contracts has not yet been fully incorporated into routine professional practice. Although nearly half of the participants (49.5%) reported using written contracts, only 37.7% stated that they always do so. Moreover, 36.8% reported that they never individualize their contracts. Another important finding concerns the absence of two witness signatures in the contracts executed, a basic requirement for their legal validity. According to the results, 66.3% of the participants reported that they never obtain these signatures. In addition, a substantial proportion of professionals reported that they never or only rarely inform patients that the expected improvement during treatment will be exclusively aesthetic (42.9%) or exclusively functional (44.9%).

**Table 7:** Dentists’ vulnerability to legal actions in orthodontic practice according to contractual practices and written information provided.

Contractual practices	Always n (%)	Often n (%)	Sometimes n (%)	Rarely n (%)	Never n (%)
Written treatment contracts	93 (37.7)	32 (13.0)	32 (13.0)	38 (15.4)	52 (21.1)
Individual treatment contracts	51 (20.6)	15 (6.1)	42 (17.0)	48 (19.4)	91 (36.8)
Contracts specifying treatment duration	65 (26.3)	33 (13.4)	38 (15.4)	35 (14.2)	76 (30.8)
Contracts specifying treatment fees	94 (38.1)	27 (10.9)	30 (12.1)	43 (17.4)	53 (21.5)
Explanation of contractual clauses to patients	89 (36.0)	23 (9.3)	42 (17.0)	42 (17.0)	51 (20.6)
Contracts including treatment technique	47 (19.0)	17 (6.9)	44 (17.8)	44 (17.8)	95 (38.5)
Contracts signed by two witnesses	23 (9.3)	7 (2.8)	15 (6.1)	38 (15.4)	163 (66.3)
Professional liability insurance contracts	54 (21.9)	7 (2.8)	15 (6.1)	18 (7.3)	163 (61.9)
Improvement exclusively functional	55 (22.3)	29 (11.7)	52 (21.1)	64 (25.9)	47 (19.0)
Improvement exclusively aesthetic	46 (18.6)	43 (17.4)	52 (21.1)	42 (17.0)	65 (25.9)

The results presented in Table 8 indicate that written documentation confirming the patient’s awareness of treatment stages is seldom incorporated into professional practice. This includes written records regarding the need for patient cooperation, instructions on retainers and extraoral appliances, and monitoring of appointment attendance, which were reported as never or rarely performed by 28.3%, 45.8%, and 18.3% of respondents, respectively.

**Table 8:** Frequency of written instructions provided by dentists to their patients.

Written patient instructions	Always n (%)	Often n (%)	Sometimes n (%)	Rarely n (%)	Never n (%)
Appointment attendance control	179 (72.5)	15 (5.9)	0	12 (4.9)	33 (13.4)
Instructions about appliances/retainers	87 (35.2)	11 (4.4)	31 (12.6)	56 (22.7)	62 (23.1)
Cooperation attitudes for treatment success	95 (38.5)	30 (12.1)	37 (15.0)	45 (18.2)	40 (16.2)
Need for patient cooperation	119 (48.2)	27 (10.9)	31 (12.6)	30 (12.1)	40 (16.2)

Figure 1 presents the main diagnostic records requested by professionals for orthodontic treatment. All participating professionals reported requesting initial orthodontic documentation, and the vast majority (87.0%) stated that they consistently require these records. Furthermore, 77.7% reported archiving all patient records even after completion of orthodontic treatment.

**Figure 1:**
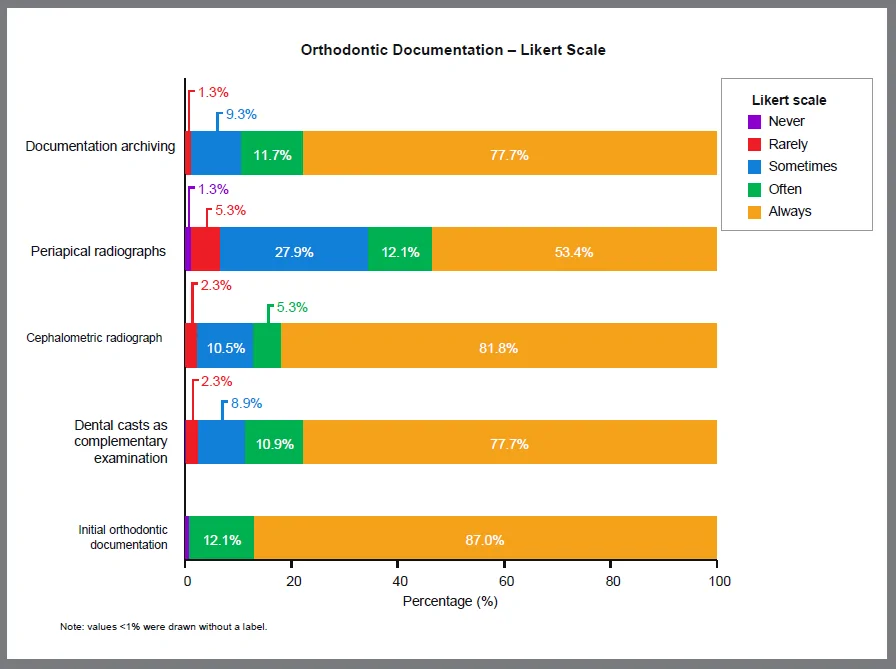
Orthodontic documentation - Likert scale.

With respect to the post-treatment phase (Fig. 2), 56.7% of respondents reported that they never documented, in writing, the patient’s degree of satisfaction at the end of orthodontic treatment, whereas 33.2% reported that they never obtained written acknowledgment or receipt from patients regarding the recommendations provided to them.

**Figure 2:**
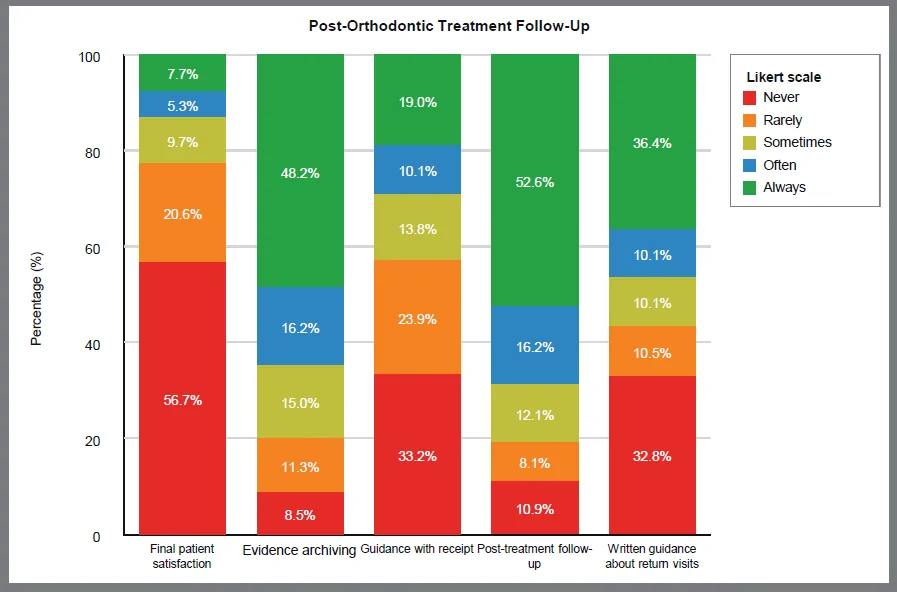
Post-orthodontic treatment follow-up.

Figure 3 illustrates the level of concern reported by the interviewed professionals regarding legal aspects related to the exercise of their profession.

**Figure 3:**
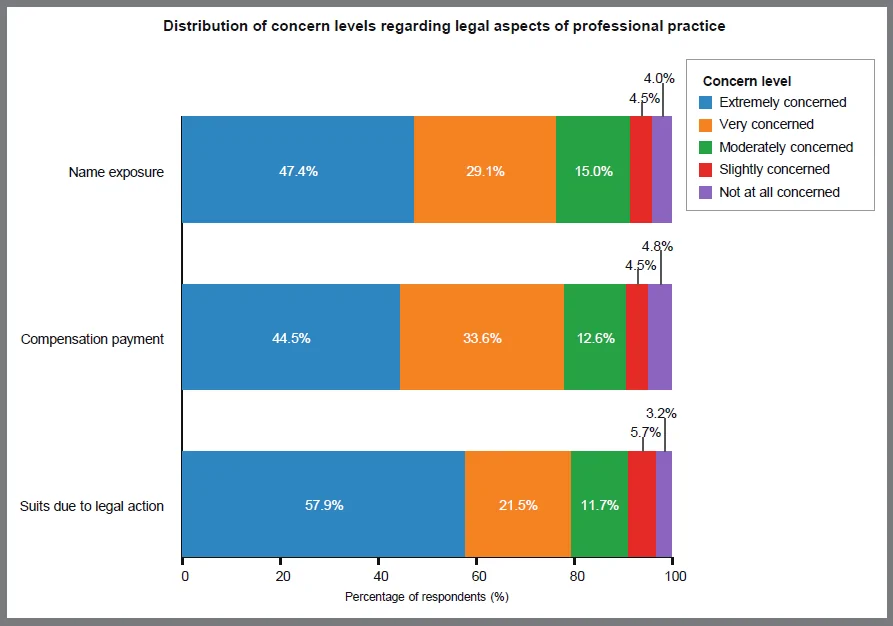
Distribution of concern levels regarding legal aspects of professional practice.

## DISCUSSION

With the expansion of mechanisms for consumer protection and the growing awareness of the population regarding their rights, there has been an increase in the filing of legal actions brought by dissatisfied patients against dental practitioners.^18^ Until the study conducted by Guedes et al.,^19^ the literature lacked an instrument capable of measuring the potential risk of involvement of professionals practicing Orthodontics in Brazil in indemnity-related legal disputes, despite the fact that this dental specialty has been frequently involved in litigation.^22^ ^-^ ^24^ The questionnaire developed and validated by those authors proved to be a reliable instrument for assessing the risks to which orthodontic professionals are exposed in the course of their practice.^19^

Regarding respondents’ legal vulnerability profile, non-specialists presented greater risks, compared with specialists, with no significant differences observed according to the institution responsible for postgraduate training, highlighting the importance of specialization in professional practice. Considering both the time since graduation in Dentistry and the duration of postgraduate training, the results indicated that professionals with fewer years of clinical experience were exposed to greater risks, suggesting that professional experience influences not only exposure to legal claims but also the adoption of practices that contribute to safer professional practice. In addition, a statistically significant difference was observed when legal vulnerability was analyzed according to academic background, with professionals who graduated from private institutions showing greater exposure. Although the findings of the present study suggest greater vulnerability among professionals with fewer years of practice, a study conducted in South Korea^25^ reported a higher frequency of accidents and legal disputes among more experienced orthodontists, particularly those aged between 40 and 50 years. The authors attributed these findings to greater clinical exposure and the larger cumulative number of patients treated throughout their careers, rather than to lower professional competence. Therefore, professional experience appears to play a dual role, contributing to the adoption of safer practices, while simultaneously increasing the likelihood of involvement in litigation as a consequence of greater exposure to clinical practice.

The literature shows that a substantial proportion of legal actions arise from failures in the professional-patient relationship.^26^ ^-^ ^28^ According to the professionals interviewed in the present study, the patient’s level of education, profession, and economic status are factors that may directly influence the professional-patient relationship and, consequently, the occurrence of legal claims. As shown in Table 2, only 0.8% to 3.6% of participants strongly disagreed that these factors may be determinants in the occurrence of legal proceedings.

The rules governing dental treatment and the professional-patient relationship are based on the Brazilian CPC, since this relationship is characterized as a consumer relationship in which the patient is regarded as the consumer of the service provided by the professional.^29^ ^-^ ^31^ Nevertheless, the present study showed that 48.1% of professionals were unaware of this information, and only 18.6% of participants agreed that professional liability is governed by the CPC. This lack of knowledge may be related to deficiencies in professional training with respect to basic legal responsibilities associated with professional practice, as reflected in the findings presented in Table 3.

The Brazilian CPC was enacted with the purpose of protecting the patient/consumer, who is regarded as the most vulnerable party in the consumer relationship.^9^ ^-^ ^11^ For this reason, in civil liability claims involving dissatisfied patients, the burden of proof may be shifted in order to facilitate the patient’s defense, given the patient’s limited technical capacity to assess the treatment received^30^ ^-^ ^32^. Accordingly, professionals must always be able to demonstrate the absence of fault with respect to the harm alleged by the patient, which underscores the importance of proper orthodontic documentation.^32^ According to the findings of the present study, 75.8% of respondents reported that they possessed documentary evidence in the event of legal proceedings. However, 57.9% incorrectly believed that fault should be proven by the dissatisfied patient, and 42.5% were unaware that their professional activity may be characterized as involving an obligation of result.

Although previous studies had already shown that a large proportion of interviewed professionals use some type of contract in their treatments,^22^ ^,^ ^32^ those studies involved very small samples^22^ or did not address how such contracts were actually developed.^32^ In the present study, although 93 professionals reported always using written contracts, corresponding to 37.7% of the sample, these contracts were generally not prepared with specialized legal guidance, as 70.8% reported lacking such support in their professional practice. Furthermore, these contracts were not developed on an individualized basis, since more than half of the professionals (56.2%) reported that they never or only rarely individualized their contracts. In addition, 49.0% of participants stated that their contracts primarily addressed issues related to treatment costs and fees. These findings, together with data from the literature,^22^ suggest that professionals have limited knowledge regarding the types of evidence that effectively hold legal validity. Nevertheless, with respect to basic treatment instructions - such as appointment attendance control and the need for patient cooperation during treatment, including the use of extraoral appliances -, professionals reported the habit of maintaining written records.

Of the 247 professionals interviewed in the present study, 192 (77.0% of the sample) reported archiving the patient’s orthodontic records at the end of treatment. Similarly, in the study conducted by Barroso et al.,^23^ 254 participants (75.3% of the sample) reported retaining such documentation for 20 years or longer. This practice is important as a means of safeguarding the professional in the event of a future legal action brought by the patient.^23^ The maintenance of complete, structured, and properly archived dental records constitutes the most effective means of protecting the professional against legal claims.^31^ According to the study by Melani and Silva,^32^ in the context of legal proceedings, only 20% of respondents considered the existence of a written contract to be the most important element, whereas 60% considered a complete clinical record to be more important. In the present study, the authors also investigated whether professionals documented the patient’s degree of satisfaction at the end of treatment and obtained the patient’s signature; however, 67.3% of respondents reported that they did not have this practice.

With respect to the responses obtained through the questionnaire, the findings indicate that professionals remain vulnerable in terms of their knowledge of the legal consequences of their own professional activity. In addition, they appeared to be markedly negligent with regard to contractual relationships, which were only rarely established in an individualized manner, and especially with regard to other phases of treatment, such as written control of appointment attendance and formal documentation of the patient’s awareness of treatment-related issues. Such practices confirm the professional vulnerability of these practitioners, both in terms of preventing legal claims and in terms of being able to respond adequately when such claims arise. Although many respondents did not appear to be fully aware of this lack of preparedness, they nevertheless expressed concern regarding the possible negative consequences of becoming involved in legal proceedings, particularly the stress arising from litigation, the exposure of their name, and the payment of financial compensation to the patient.

## CONCLUSION

Within the limitations of this cross-sectional study, orthodontic practitioners showed vulnerability to compensation-related lawsuits. In the studied sample, higher judicial vulnerability scores were associated with lack of specialization, shorter time since graduation and specialization, and absence of legal guidance in professional practice. These findings suggest the importance of professional qualification and legal awareness in Orthodontics, and should be interpreted in light of the observational design of the study.

## Data Availability

Data Availability Statement: Due to the sensitive nature of the data and ethical restrictions, the datasets supporting this study are not publicly available.
